# Circadian dimensions in insomnia disorder: mechanistic evidence, candidate phenotypes, and a phenotype-stratified research framework

**DOI:** 10.3389/fpsyt.2026.1884632

**Published:** 2026-07-17

**Authors:** Shasha Zhang, Yan Liu, Jinzhi Tan, Wenting Si, Hongyi Liu, Yini Huang, Yanfang Li, Jianfen Xiao

**Affiliations:** Changsha Hospital of Traditional Chinese Medicine (Changsha Eighth Hospital), Changsha, China

**Keywords:** circadian rhythm, cognitive behavioral therapy for insomnia, hyperarousal, insomnia disorder, light therapy, melatonin, precision sleep medicine, sleep homeostasis

## Abstract

Insomnia disorder is one of the most common sleep disorders and is traditionally conceptualized in terms of hyperarousal, altered sleep homeostasis, and cognitive-behavioral perpetuating factors. The two-process model of sleep regulation indicates that sleep initiation and maintenance depend not only on homeostatic sleep pressure but also on circadian phase, amplitude, and stability. Evidence suggests that some patients presenting with insomnia complaints have misalignment between endogenous circadian phase and the intended sleep window, whereas others show reduced rest-activity amplitude, increased sleep-timing variability, or abnormal light exposure patterns. This narrative review integrates clinical, measurement, mechanistic, and intervention evidence on circadian dimensions in insomnia disorder and proposes a candidate circadian phenotyping framework for future validation. The framework combines sleep diaries, actigraphy, light exposure assessment, dim-light melatonin onset (DLMO), and core body temperature rhythms to distinguish circadian rhythm sleep-wake disorder (CRSWD)-dominant insomnia complaints, insomnia disorder with circadian modifiers, and comorbid insomnia disorder and CRSWD. Cognitive behavioral therapy for insomnia (CBT-I) remains first-line treatment. Fixed wake time, morning light, evening light restriction, timed low-dose melatonin, scheduled activity, and multicomponent approaches are positioned as candidate adjunctive modules to be tested in phenotype-stratified trials. These phenotypes are not diagnostic categories, clinical decision algorithms, or treatment guidelines. The review instead translates circadian evidence in insomnia into testable propositions, including provisional phenotype definitions, recommended measurement strategies, falsifiable predictions, and future trial designs. Operational thresholds, reproducibility, predictive validity, incremental treatment benefit, optimal parameters, safety, implementation feasibility, and cost-effectiveness require prospective phenotype-stratified randomized trials and long-term follow-up.

## 1 Introduction

Insomnia disorder is characterized by persistent difficulty initiating sleep, difficulty maintaining sleep, or early morning awakening despite adequate sleep opportunity, together with daytime impairment (1–3). Chronic insomnia symptoms are common in adults and are particularly prevalent among people with depressive, anxiety, and psychotic disorders (4). Longitudinal studies and meta-analyses further indicate that persistent insomnia is associated with increased risk of subsequent depression, anxiety, and other mental disorders (5, 6). Clarifying mechanistic heterogeneity in insomnia is therefore relevant not only to sleep outcomes but also to mental health prevention and early intervention.

Traditional models of insomnia emphasize hyperarousal, impaired sleep homeostasis, and cognitive-behavioral perpetuating factors. Hyperarousal models propose that insomnia involves sustained cortical, autonomic, cognitive-emotional, and neuroendocrine activation that interferes with the transition into and maintenance of sleep (7, 8). However, the two-process model indicates that the opening and closing of the sleep window depend not only on homeostatic sleep pressure but also on circadian output (8). When circadian wake-promoting signals remain high at the intended bedtime, or when endogenous biological night is misaligned with social schedules, patients may experience difficulty initiating sleep, difficulty maintaining sleep, or early morning awakening even when sleep pressure has accumulated.

Insomnia complaints also overlap with circadian rhythm sleep-wake disorders (CRSWD). Flynn-Evans and colleagues found that a substantial subset of patients meeting criteria for primary insomnia attempted sleep at an unfavorable circadian phase, suggesting that some apparent insomnia may reflect unrecognized phase-angle abnormalities (9). A more recent insomnia disorder study using DLMO, sleep diaries, and actigraphy also showed measurable associations between endogenous circadian timing and sleep symptoms (10). The American Academy of Sleep Medicine guideline for CRSWD further emphasizes that delayed sleep-wake phase disorder (DSWPD) and advanced sleep-wake phase disorder (ASWPD) may present with insomnia-like complaints such as sleep-onset difficulty, early awakening, and daytime dysfunction (11). Discussion of circadian dimensions in insomnia must therefore address diagnostic boundaries, comorbidity, and symptom overlap.

Objective measurement studies further suggest that some patients with insomnia may show delayed sleep timing, reduced rest-activity amplitude, increased sleep-timing variability, or abnormal light exposure patterns (12–14). Modern light exposure patterns, characterized by insufficient daytime light and excessive evening or sleep-period light, may flatten light-dark contrast, weaken circadian amplitude, and promote phase delay (15–21). Naturalistic and micro-longitudinal studies also suggest that insufficient outdoor light and greater sleep-period light exposure are associated with sleep-wake fragmentation, poorer sleep continuity, and later sleep timing (22, 23). These findings do not imply that circadian disruption is a unified cause of insomnia disorder. Instead, circadian factors may function as modifiers of symptom maintenance and treatment response in selected patients.

### 1.1 Review approach and evidence appraisal

This article is a narrative review that develops a phenotype-stratified research framework rather than a systematic review, scoping review, clinical guideline, or diagnostic algorithm. We searched PubMed, Web of Science, and Google Scholar from database inception to April 2026. Search terms included insomnia disorder, chronic insomnia, circadian rhythm, circadian misalignment, dim light melatonin onset, core body temperature, actigraphy, light exposure, light therapy, melatonin, chronopharmacology, cognitive behavioral therapy for insomnia, hyperarousal, delayed sleep-wake phase disorder, sleep regularity, social jetlag, and wearable sleep monitoring. Priority was given to clinical guidelines, systematic reviews, meta-analyses, randomized trials, mechanistic studies, and representative observational studies directly relevant to the review questions. Case reports, non-peer-reviewed sources, studies with weak relevance to insomnia disorder, and studies not addressing sleep or circadian measurement were not used as major evidence sources.

Because this is a narrative review, we did not conduct protocol registration, independent dual screening, formal risk-of-bias assessment, standardized certainty grading, or quantitative synthesis. The conclusions should therefore be interpreted as a transparent narrative synthesis rather than as evidence-based clinical recommendations. To increase transparency, evidence strength was appraised narratively according to four criteria: (1) whether evidence came directly from individuals meeting criteria for insomnia disorder rather than only from CRSWD, healthy circadian experiments, or psychiatric comorbidity samples; (2) whether studies included objective circadian measures such as DLMO, core body temperature rhythm, actigraphy, or light exposure; (3) whether evidence came from randomized trials, systematic reviews, or clinical guidelines rather than cross-sectional associations or mechanistic inference alone; and (4) whether findings were replicated and linked to clinical outcomes. On this basis, we classify conclusions as stronger evidence, moderate evidence, or hypothesis-stage evidence. This appraisal was intended to make the inferential distance of each claim explicit. In particular, several propositions in this review remain partly extrapolated from CRSWD research, experimental circadian studies in healthy adults, actigraphy-based observational work, and psychiatric-comorbidity samples, whereas direct studies in rigorously defined insomnia disorder cohorts remain limited.

## 2 Clinical presentations and diagnostic boundaries: avoiding misclassification of CRSWD as insomnia phenotypes

Studying circadian dimensions in insomnia first requires a clear distinction between insomnia disorder and CRSWD. CRSWD is defined by persistent misalignment between endogenous circadian timing and required social, occupational, or educational schedules, whereas insomnia disorder requires ongoing sleep initiation or maintenance difficulty despite adequate sleep opportunity and an appropriate environment (2, 11). If a patient sleeps substantially better and longer on a self-selected schedule but experiences insomnia-like complaints when following social schedules, CRSWD or insomnia with circadian phase misalignment should be considered before assigning a diagnosis of typical insomnia disorder.

From a research perspective, samples enrolled under an insomnia label may include unrecognized CRSWD. This can exaggerate the role of circadian factors in typical insomnia disorder and complicate interpretation of intervention studies. We therefore propose distinguishing three clinical contexts: (1) CRSWD-dominant insomnia complaints, in which phase misalignment is the primary problem; (2) insomnia disorder with circadian modifiers, in which the patient meets diagnostic criteria for insomnia disorder but light exposure, sleep-wake scheduling, or rhythm stability influences symptom maintenance; and (3) comorbid insomnia disorder and CRSWD, in which chronic phase misalignment and conditioned hyperarousal are mutually reinforcing. The candidate framework proposed in this review applies primarily to the second and third contexts; the first should be managed primarily according to CRSWD principles. The proposed clinical contexts, candidate interventionmodules,candidatephenotypes,testable propositions, and evidence-confidence ratings are summarized in **Tables 1**–**5**.

**Table 1:** Three clinical contexts of circadian factors in insomnia-like complaints.

Clinical context	Primary problem/characteristics	Response to free schedule	Clinical management & priority
CRSWD-dominant insomnia complaints	Mismatch between endogenous phase and social time; should not be simply classified as an insomnia phenotype.	Sleep duration and quality improve substantially.	CRSWD treatment should be prioritized.
Insomnia disorder with circadian modifiers	Meets criteria for insomnia disorder, but light exposure, sleep-wake scheduling, or rhythm stability contributes to symptom maintenance.	Sleep may partially improve, but insomnia symptoms persist.	CBT-I remains the foundation, with circadian factors as candidate adjunctive targets.
Comorbid insomnia disorder and CRSWD	Phase misalignment coexists with conditioned arousal, rumination, or impaired sleep perception.	Free-schedule sleep improves incompletely.	Both CRSWD and insomnia-maintaining factors require attention; modular combined approaches are most relevant.

CRSWD, circadian rhythm sleep-wake disorders; CBT-I, cognitive behavioral therapy for insomnia.

**Table 2:** Candidate circadian-related intervention modules in insomnia disorder: possible use, risks, and current evidence position.

Module	Candidate use	Main risks	Evidence level	Current position
CBT-I	Chronic insomnia disorder; especially hyperarousal and behavioral perpetuating factors	Transient sleepiness, adherence issues; sleep restriction may impair daytime safety	Strong	First-line treatment
Fixed wake time	Rhythm instability, delayed timing, excessive time in bed	Short-term sleep-loss sensation and daytime sleepiness	Moderate	Core behavioral time anchor within CBT-I and circadian research
Morning bright light	Delayed phase, morning sleepiness, low daytime light	Eye discomfort, headache, agitation, bipolar-spectrum risk	Moderate	Candidate adjunctive module requiring phenotype-stratified validation
Evening light	Objectively supported advanced phase or selected early awakening presentations	Worsened sleep-onset difficulty, incorrect phase shifting	Limited	Use cautiously only with objective evidence of advanced phase
Timed low-dose melatonin	Delayed-phase sleep-onset complaints	Next-day sleepiness, incorrect timing, product variability	Moderate	Not a general hypnotic; requires circadian timing and validation
Scheduled exercise/activity	Reduced amplitude, low daytime activity	Late high-intensity exercise may interfere with sleep onset	Moderate to limited	Adjunctive lifestyle module
Wearable monitoring	Phenotyping, longitudinal follow-up	Algorithm error, privacy, uncertain phase estimation	Developing	Adjunctive assessment tool; not a DLMO substitute
Closed-loop interventions	Research stage	Black-box algorithms, safety uncertainty	Weak	Research stage; not recommended for routine care

CBT-I, cognitive behavioral therapy for insomnia; DLMO, dim light melatonin onset.

**Table 3:** Candidate circadian-related phenotypes in insomnia disorder: identification clues and testable intervention hypotheses.

Candidate phenotype	Clinical clues	Supportive measures	Candidate objective parameters	Testable intervention hypothesis	Evidence status
Delayed phase	Sleep-onset difficulty; late sleep and wake times; catch-up sleep; morning sleepiness	Diary, actigraphy, DLMO, chronotype, light exposure	DLMO timing; DLMO-to-target-bedtime phase angle; free-day sleep midpoint; workday-free day sleep midpoint difference; actigraphic sleep onset and wake timing; morning sleepiness	Fixed wake time; morning light; evening light restriction; timed low-dose melatonin; CBT-I stimulus control	Moderate: supported by CRSWD and insomnia DLMO/phase-angle studies; phenotype-stratified insomnia RCTs lacking
Advanced phase	Early morning awakening; early evening sleepiness; advanced sleep window	Diary, actigraphy, DLMO, core temperature nadir	Advanced DLMO; core body temperature nadir; early sleep midpoint; early evening sleepiness; early morning awakening pattern; assessment of depression, aging-related sleep change, medications, and COMISA	Bedtime adjustment; avoid too-early bedtime; cautious evening light only with objective evidence; assess depression/aging	Limited: extrapolated from phase models, aging, and early awakening; objective phase confirmation needed
Reduced amplitude	Low daytime activity; relatively high nocturnal activity; light sleep; fatigue	Relative amplitude, daytime light, nocturnal activity, melatonin/cortisol rhythm	Actigraphic relative amplitude; daytime activity level; nocturnal activity counts; daytime light exposure; sleep-period light exposure; optional melatonin, cortisol, or temperature rhythm amplitude	Daytime light enhancement; scheduled activity/exercise; behavioral activation; fixed wake time	Limited to moderate: association evidence; causality and incremental treatment benefit uncertain
Unstable rhythm	Variable sleep onset/wake time; workday-free day differences; social jetlag	Diary, actigraphy, workday-free day differences, nap recording, SRI or related metrics	Sleep midpoint variability; Sleep Regularity Index; interdaily stability; intradaily variability; social jetlag; catch-up sleep frequency; nap timing and duration	Stable wake time; reduce catch-up sleep; limit long naps; CBT-I sleep-window adjustment.	Moderate: social jetlag, actigraphy, and regularity evidence; causality insufficient
Hyperarousal-dominant	Rumination in bed; impaired sleep perception; anxiety; conditioned insomnia	ISI, PSQI, diary, PSG to exclude other disorders, psychiatric measures	Insomnia Severity Index; Pittsburgh Sleep Quality Index; sleep-related rumination; pre-sleep arousal; diary-actigraphy or diary-PSG discrepancy; psychiatric symptom assessment; medication review; exclusion or stratification of other sleep disorders	Core CBT-I, cognitive therapy, relaxation, psychiatric comorbidity management	CBT-I evidence strong; incremental value of circadian modules unclear
Mixed phenotype	Phase misalignment, rhythm instability, hyperarousal, or psychiatric comorbidity coexist	Diary, actigraphy, light exposure, DLMO, psychiatric assessment	Combined assessment of phase, amplitude, regularity, hyperarousal, psychiatric symptoms, COMISA risk, medications, substance use, and other sleep disorders	Modular strategy ordered by dominant and safest targets	Hypothesis stage; prospective stratification needed

CBT-I, cognitive behavioral therapy for insomnia; CRSWD, circadian rhythm sleep-wake disorders; DLMO, dim light melatonin onset; ISI, Insomnia Severity Index; PSG, polysomnography; PSQI, Pittsburgh Sleep Quality Index; RCTs, randomized controlled trials; SRI, Sleep Regularity Index; COMISA, comorbid insomnia and sleep apnea.

**Table 4:** Testable propositions, evidence gaps, and suggested designs for phenotype-stratified insomnia trials.

Proposition	Testable prediction	Recommended measures	Falsification criterion	Main evidence gap	Suggested trial design
Delayed-phase patients are more likely to benefit from phase-advancing modules	Greater DLMO delay predicts larger improvements in sleep latency and sleep midpoint after CBT-I plus morning light/timed melatonin	DLMO, sleep midpoint, ISI, actigraphy	DLMO delay does not predict response, or phase advance is unrelated to symptom improvement	Lack of insomnia trials enrolling by objective DLMO delay	Randomize DLMO-delayed insomnia patients to CBT-I vs CBT-I plus morning light/timed melatonin
Unstable-rhythm patients are more likely to benefit from behavioral time anchoring	Reduced sleep midpoint variability mediates improvements in ISI and daytime functioning	Diary, actigraphy, social jetlag, SRI, daytime functioning	Regularity improvement is unrelated to clinical outcomes	Longitudinal intervention evidence is limited	Randomize high-variability patients to CBT-I vs CBT-I plus fixed wake time and behavioral time anchors
Reduced-amplitude patients may require daytime zeitgeber enhancement	Daytime light/activity increases relative amplitude and improves fatigue and sleep continuity	Light sensor, relative amplitude, fatigue measures	Amplitude does not change or does not predict symptom improvement	Causal role of amplitude in subjective sleep and daytime function is unclear	Randomize low-amplitude/low-light patients to CBT-I vs CBT-I plus daytime light/activity module
Hyperarousal-dominant patients may show limited incremental benefit from circadian modules	Core CBT-I improves rumination, conditioned arousal, and insomnia severity more than circadian modules alone	ISI, PSQI, rumination/anxiety measures, PSG	Circadian modules show independent benefit despite no phase evidence	Few trials compare CBT-I core components with circadian modules	In patients without phase misalignment, compare CBT-I core modules, circadian modules, and combined treatment

CBT-I, cognitive behavioral therapy for insomnia; DLMO, dim light melatonin onset; ISI, Insomnia Severity Index; PSG, polysomnography; PSQI, Pittsburgh Sleep Quality Index; SRI, Sleep Regularity Index.

**Table 5:** Direct evidence, extrapolated evidence, and current confidence for key claims.

Claim	Direct insomnia evidence	Extrapolated evidence	Confidence	Main limitation
Some insomnia-like complaints are related to phase misalignment	Flynn-Evans phase-angle study; insomnia DLMO study	CRSWD guidelines; phase response studies	Moderate	Possible inclusion of unrecognized CRSWD
Actigraphy can identify rhythm instability	Insomnia/psychiatric samples with sleep-timing variability	Social jetlag, SRI, and regularity research	Moderate	Behavioral rhythm is not endogenous phase
Morning light/melatonin may help delayed-phase patients	Direct insomnia RCTs limited	DSWPD and healthy adult phase-shifting studies	Moderate-low	Lack of phenotype-stratified insomnia RCTs
Reduced rhythm amplitude may relate to fatigue and light sleep	Mainly actigraphy and association studies	Light, activity, and psychiatric comorbidity literature	Limited	Causality unclear
Hyperarousal-dominant patients may have limited circadian-module gain	Strong CBT-I evidence for hyperarousal/behavioral factors	Mechanistic inference	Limited	Incremental module trials lacking
Wearables can assist rhythm assessment	Useful for activity and sleep regularity monitoring	Digital sleep technology validation studies	Developing	Cannot replace DLMO or core temperature rhythm
COMISA and psychiatric disorders may confound circadian phenotype assignment	COMISA and affective symptoms frequently overlap with insomnia complaints and sleep fragmentation	OSA, depression, bipolar-spectrum disorders, anxiety disorders, psychotropic medications, and substance use can alter sleep timing, sleep continuity, activity rhythms, and treatment response	Moderate	Few phenotype-stratified insomnia studies have systematically separated circadian mechanisms from COMISA, active mood episodes, medication effects, and other sleep disorder contributors

CBT-I, cognitive behavioral therapy for insomnia; CRSWD, circadian rhythm sleep-wake disorders; DLMO, dim light melatonin onset; DSWPD, delayed sleep-wake phase disorder; RCTs, randomized controlled trials; SRI, Sleep Regularity Index; COMISA, comorbid insomnia and sleep apnea; OSA, obstructive sleep apnea.

Free-day sleep is an important diagnostic clue. Marked improvement in sleep duration and quality during weekends, holidays, or unrestricted schedules suggests misalignment between biological and required external time. Conversely, persistent sleep-onset difficulty, repeated awakenings, or impaired sleep perception even within a biologically appropriate sleep window suggests psychophysiological insomnia, hyperarousal, psychiatric symptoms, or other sleep disorders. In addition to sleep diaries and actigraphy, DLMO, core body temperature nadir, and light exposure recording can help clarify the relationship between endogenous phase and behavioral timing (10, 24–26). In studies lacking objective phase assessment or sufficient information on free-day sleep, “circadian abnormalities in insomnia disorder” should be interpreted cautiously as rhythm-related factors in insomnia-like complaints rather than intrinsic mechanisms of typical insomnia disorder. Future studies should exclude CRSWD-dominant cases at baseline or treat them as a separate stratum rather than pooling all insomnia-like presentations.

Comorbid insomnia and sleep apnea (COMISA) should also be considered as a clinically important confounder in circadian phenotyping. Patients with COMISA may report sleep-maintenance insomnia, early morning awakening, non-restorative sleep, daytime fatigue, and mood symptoms that can mimic reduced-amplitude or unstable-rhythm phenotypes. Respiratory-event-related arousals, intermittent hypoxemia, nocturia, and sleep fragmentation may alter actigraphic sleep continuity and rest-activity patterns without necessarily indicating primary circadian dysfunction. Therefore, future phenotype-stratified studies should screen for obstructive sleep apnea risk using clinical history, snoring and witnessed-apnea assessment, daytime sleepiness, cardiometabolic risk factors, and validated screening tools, with polysomnography or home sleep apnea testing considered when clinically indicated. COMISA cases should be excluded, analyzed as a separate stratum, or adjusted for in sensitivity analyses rather than being pooled uncritically with uncomplicated insomnia disorder (27).

Affective and other psychiatric disorders represent another important source of diagnostic overlap. Depressive episodes may present with early morning awakening, reduced daytime activity, fatigue, and blunted rest-activity amplitude, whereas bipolar-spectrum conditions may involve reduced sleep need, delayed or unstable sleep timing, and heightened sensitivity to light or schedule shifts. Anxiety disorders, psychotic disorders, substance use, and psychotropic medications may also influence sleep timing, sleep continuity, light exposure, activity rhythms, and treatment response. Candidate circadian phenotypes should therefore not be assigned solely on the basis of sleep timing, early awakening, or low daytime activity when active psychiatric symptoms are prominent. Future studies should include standardized assessment of depressive symptoms, anxiety symptoms, bipolar-spectrum history, current mood state, psychotropic medication use, substance use, and suicidality, and should either stratify by psychiatric comorbidity or adjust for these variables in phenotype-response analyses.

## 3 Objective circadian phenotypes and mechanistic basis

This section distinguishes relatively direct evidence from inference. Evidence on phase misalignment, light phase responses, and melatonin rhythm comes primarily from human circadian experiments, CRSWD research, and a limited number of phase-assessment studies in insomnia. Evidence on reduced circadian amplitude, hypothalamic-pituitary-adrenal (HPA) axis rhythms, rapid eye movement (REM) sleep, slow-wave sleep, and neuroplasticity is more indirect. We use the latter evidence only to frame testable mechanistic hypotheses, not to define validated clinical phenotypes, diagnostic categories, or treatment-selection rules.

Actigraphy can record rest-activity rhythms continuously in the natural environment and is a useful tool for identifying rhythm-related patterns (12, 13, 24). In chronic insomnia disorder, actigraphy-derived rest-activity and phase parameters have been associated with insomnia severity and selected polysomnographic variables (28). Current evidence suggests three common objective patterns: delayed sleep timing, reduced rest-activity amplitude, and increased day-to-day variability in sleep timing. Delayed timing is common among adolescents, young adults, and patients with sleep-onset complaints. Reduced amplitude may appear as lower daytime activity and relatively higher nocturnal activity. Unstable rhythms may appear as increased workday-free day sleep midpoint differences, catch-up sleep, social jetlag, and lower sleep regularity (14, 29–33). However, actigraphy captures behavioral rhythms rather than endogenous biological phase and should not be used alone to diagnose circadian phase misalignment.

At the mechanistic level, the circadian system consists of molecular clocks, the suprachiasmatic nucleus (SCN), and peripheral outputs. PER, CRY, CLOCK, BMAL1, and related transcription-translation feedback loops generate cellular circadian oscillations. Associations between single clock-gene variants and insomnia or chronotype are generally small and insufficient for clinical diagnosis or treatment selection (14). More clinically relevant may be circadian resilience: the capacity to maintain stable phase and amplitude despite genetic background, light exposure patterns, social timing, and stress.

**Figure 1:**
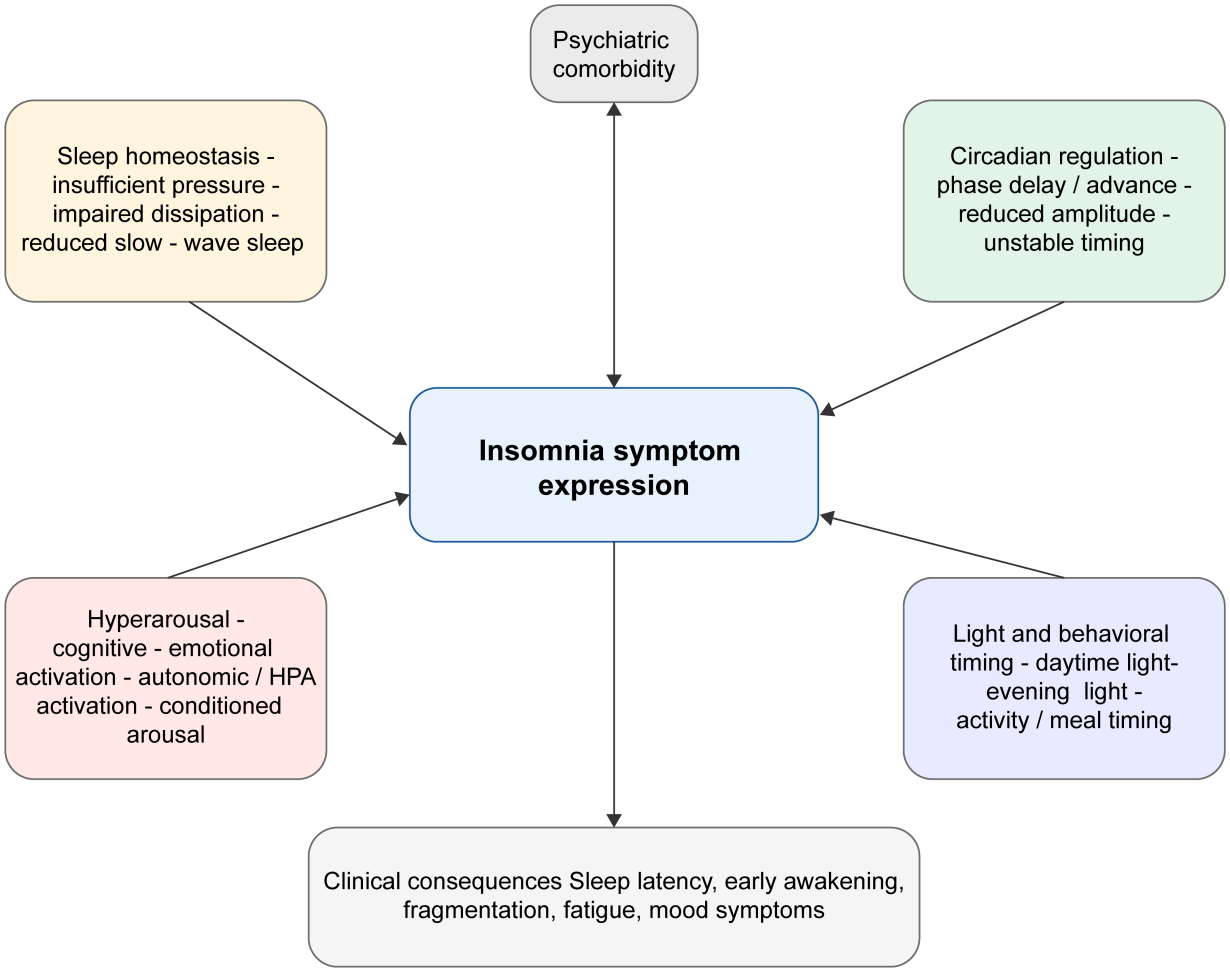
Conceptual model linking sleep homeostasis, hyperarousal, and circadian regulation in insomnia disorder. This research-oriented model illustrates how insomnia symptoms may be maintained by abnormal sleep pressure, circadian phase or amplitude disturbances, cognitive-emotional hyperarousal, abnormal light exposure, and disrupted behavioral timing cues.

**Figure 2:**
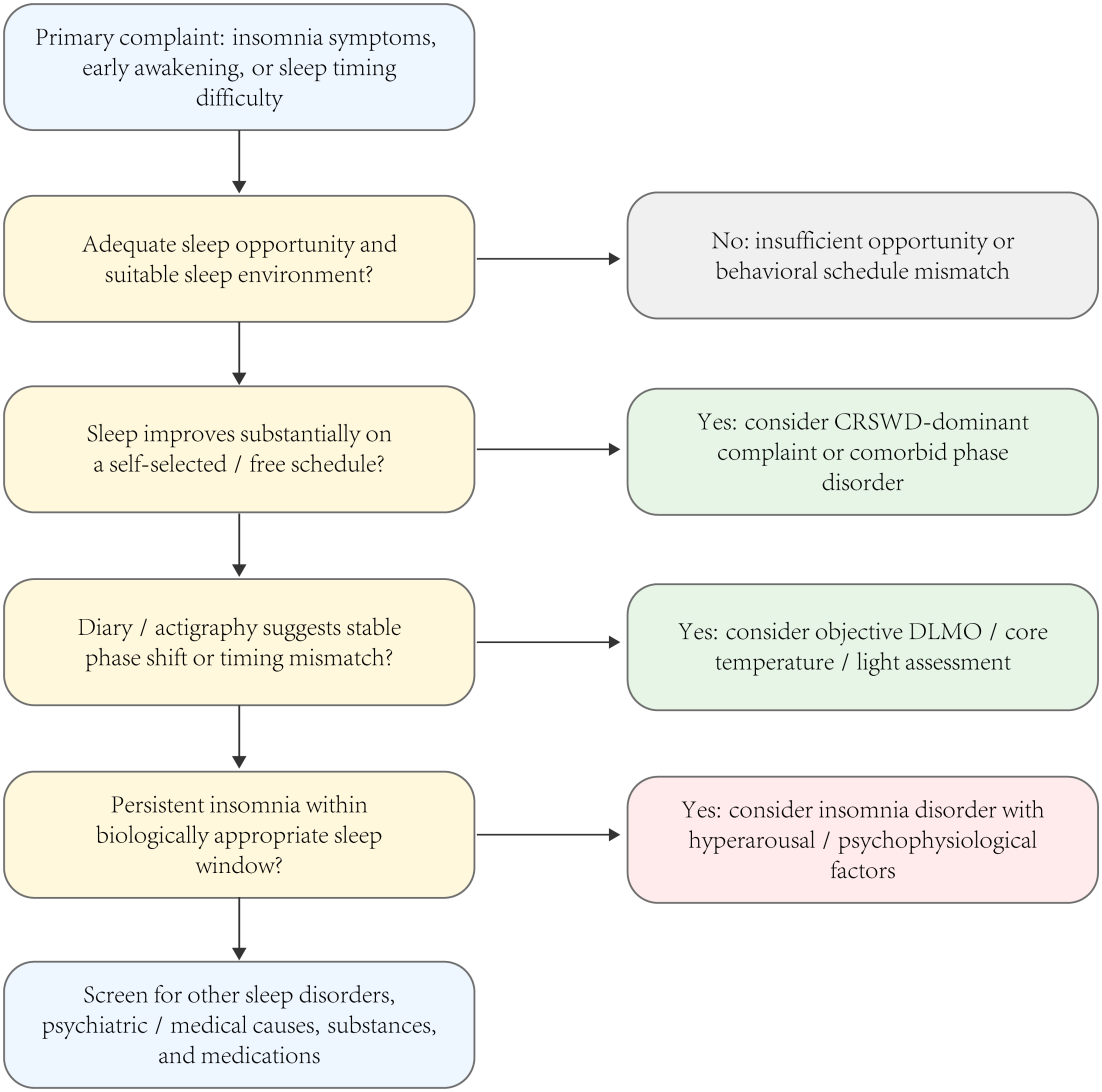
Distinguishing insomnia disorder, CRSWD, and comorbid presentations. Free-day sleep, workday-free day differences, actigraphy, DLMO, and screening for psychiatric, medical, medication, and other sleep disorder contributors can help separate typical insomnia disorder, CRSWD, comorbid presentations, and alternative explanations.

**Figure 3:**
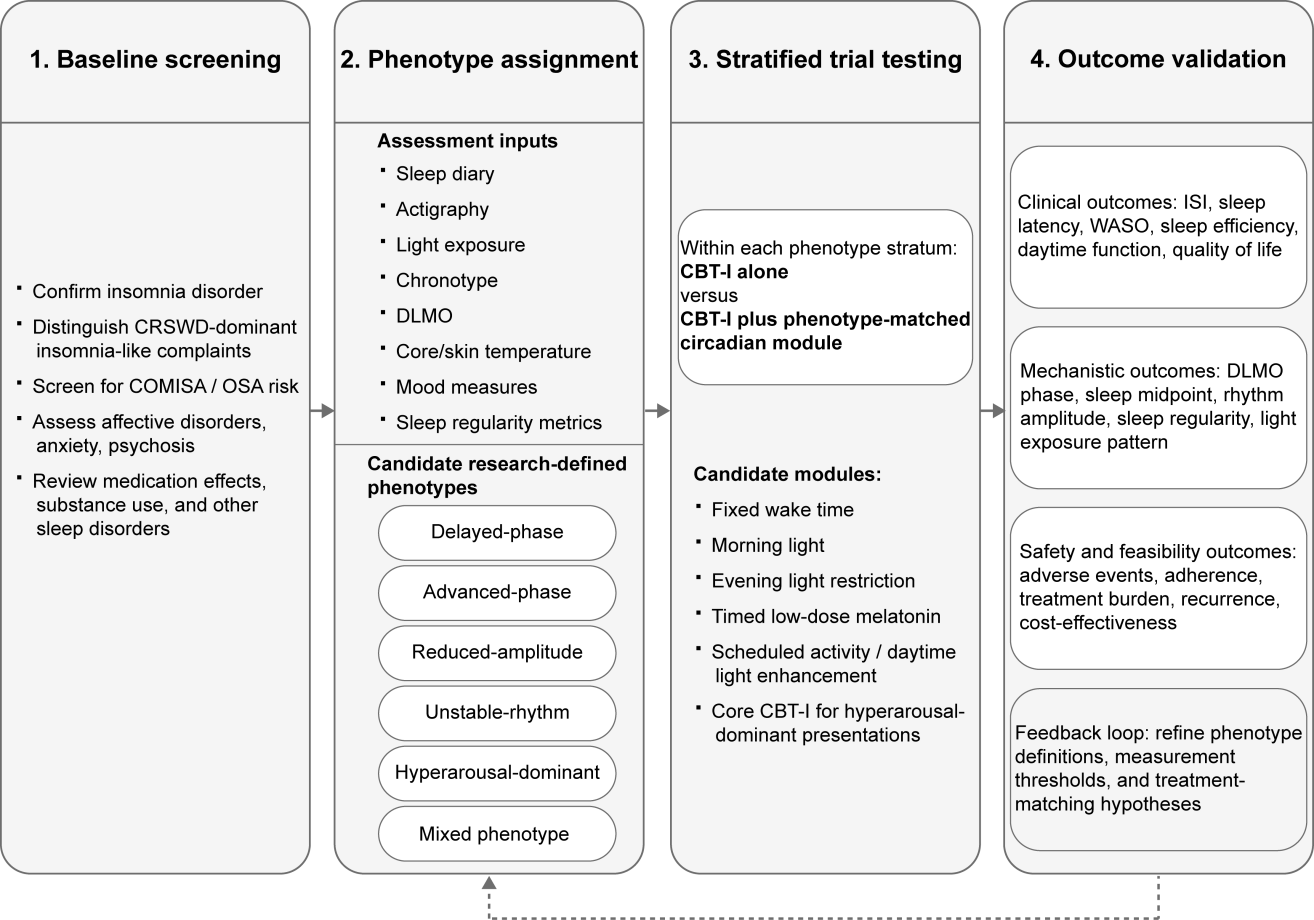
Hypothesis-generating pathway for phenotype-stratified insomnia trials. The pathway summarizes baseline screening, multi-indicator phenotype assignment, phenotype-stratified trial testing, and outcome validation. It is intended as a research framework rather than a clinical decision algorithm and requires prospective validation.

The SCN is the central circadian pacemaker. It receives input from intrinsically photosensitive retinal ganglion cells and regulates sleep-promoting and wake-promoting systems through multisynaptic pathways (15, 17). If SCN phase is misaligned with target bedtime, patients may remain within a circadian wake-promoting window despite subjective fatigue, resulting in difficulty initiating sleep. If SCN output amplitude is reduced or external entrainment signals are weak, the boundary between sleep and wake may become blurred, increasing light sleep, awakenings, and fragmentation. These pathways are biologically plausible but remain incompletely established as causal mechanisms in human insomnia disorder. The conceptual model, evaluation workflow, and phenotype-stratified research framework are shown in **Figures 1**–**3**

Light is the strongest environmental time cue. Evening or early biological night light can delay circadian phase and suppress melatonin, whereas morning light usually advances phase (15, 17, 18). Non-visual projections from ipRGCs can also influence alertness, affective reactivity, and reward-related circuits, so evening light may interfere with sleep initiation even without large measurable phase shifts (21). Individual light sensitivity varies markedly, indicating that future light interventions should not rely solely on fixed parameters (20, 21). The timing and duration of daytime light may also influence sleep-wake consolidation and rhythm amplitude, so future studies should report light intensity, timing, duration, and spectral characteristics (22, 23, 34). Observational meta-analytic evidence also links light at night with increased risk of sleep problems (35).

Hyperarousal, HPA-axis rhythmicity, REM sleep, slow-wave sleep, and neuroplasticity are best regarded as possible downstream, interacting, or moderating pathways rather than as established circadian mechanisms of insomnia disorder. Some patients with insomnia show elevated evening cortisol or reduced HPA-axis rhythm amplitude (36). The timing of REM sleep and slow-wave sleep is also influenced by biological night and sleep timing (37). Nevertheless, studies simultaneously measuring DLMO, core body temperature, high-density EEG, HPA-axis markers, and daytime outcomes in insomnia are scarce. Accordingly, the possibility that circadian misalignment contributes to REM instability, slow-wave sleep reduction, impaired synaptic homeostasis, or HPA-axis dysregulation should be interpreted as a mechanistic hypothesis that remains insufficiently validated in insomnia disorder. These markers should therefore remain exploratory mediators or moderators in future mechanistic studies, rather than routine phenotyping variables or treatment-matching criteria.

## 4 Candidate intervention modules: evidence, boundaries, and research hypotheses

CBT-I is first-line treatment for chronic insomnia disorder and is recommended by major guidelines (3, 38, 39). Its efficacy is supported by systematic review and meta-analysis (40). Established mechanisms include increasing homeostatic sleep pressure, reducing wakefulness in bed, lowering conditioned arousal, and modifying dysfunctional sleep beliefs. Fixed wake time, limiting daytime naps, and stabilizing the sleep window may indirectly strengthen behavioral time cues, but objective evidence that circadian stabilization mediates CBT-I response remains limited. Circadian interventions should therefore not replace CBT-I; they should be tested as candidate adjunctive modules for specific research-defined phenotypes.

Light-based intervention is grounded in the human phase response curve. For patients with delayed phase, late sleep timing, morning sleepiness, and workday sleep restriction, morning bright light has biological plausibility as a phase-advancing adjunct requiring testing in phenotype-stratified trials (11, 15, 18). In patients reporting early morning awakening, evening light should not be used empirically on the basis of early awakening alone. Sleep-period and evening light exposure may impair sleep continuity, so evening light restriction should be treated as a basic environmental control variable in future trials (23). Evening light should be considered only when sleep diaries, actigraphy, chronotype measures, or objective phase markers support advanced phase. Light treatment is generally acceptable but may produce eye discomfort, headache, agitation, worsening insomnia, and hypomania or mania in bipolar-spectrum patients (11).

Melatonin should not be conceptualized as a general hypnotic in insomnia disorder. Its main circadian value is timed phase shifting in delayed phase. The phase effect of exogenous melatonin depends on timing, dose, and individual circadian phase (41–43). In DSWPD, melatonin can advance phase, but in insomnia disorder without confirmed phase delay, melatonin alone may have limited and inconsistent benefit (11, 44). Melatonin receptor agonists may be pharmacologic options for selected sleep-onset complaints but should not be equated with circadian phase correction (43). Use in children, pregnancy, severe hepatic or renal disease, immune disorders, polypharmacy, and bipolar-spectrum risk requires caution.

Multicomponent approaches combine candidate modules according to dominant maintaining factors. In future phenotype-stratified research, patients with delayed phase and conditioned insomnia could be prioritized for testing fixed wake time, morning light, evening light restriction, timed low-dose melatonin, and CBT-I stimulus control. Patients with reduced rhythm amplitude and low daytime activity could be used to test daytime light enhancement, scheduled activity, behavioral activation, and sleep-timing anchoring. Patients with highly irregular schedules could be used to test fixed wake time, reduction of catch-up sleep, and limitation of long daytime naps. Patients dominated by hyperarousal and rumination should continue to receive core CBT-I, cognitive therapy, relaxation, and treatment of psychiatric comorbidity. The Transdiagnostic Intervention for Sleep and Circadian Dysfunction (TranS-C) illustrates a modular cross-diagnostic approach and has randomized and implementation evidence in serious mental illness and community mental health settings, but direct evidence in uncomplicated insomnia disorder remains limited (45–47).

Time-restricted eating, scheduled exercise, non-invasive brain stimulation, and wearable closed-loop interventions have mechanistic plausibility but are not ready for routine insomnia care. Regular daytime meals, avoidance of late-night eating, and appropriately timed daytime exercise may be reasonable lifestyle targets. Non-invasive stimulation and closed-loop approaches should remain within mechanistic or early translational research. Wearable devices, light sensors, skin temperature, and heart-rate variability enable long-term monitoring and model-based circadian phase estimation, but model accuracy varies across inputs and populations, and these tools cannot yet replace DLMO or validated core temperature rhythms (48, 49).

Safety should be a core outcome in trials of circadian candidate modules. Sleep restriction and fixed wake time may transiently increase daytime sleepiness and should be used cautiously in safety-sensitive occupations, driving, high fall risk, epilepsy, severe depression, or suicidal risk. Light therapy requires careful monitoring for hypomania or mania in bipolar-spectrum patients. Melatonin use should account for timing, next-day sleepiness, product dose variability, drug interactions, and limited evidence in special populations. Future trials should systematically record adverse events, adherence, treatment burden, and functional safety outcomes rather than reporting sleep outcomes alone.

## 5 Candidate phenotypes, evidence matrix, and phenotype-stratified research roadmap

On the basis of the preceding evidence, we propose a candidate circadian phenotyping framework. This is neither a diagnostic system nor a clinical guideline. Its purpose is to help researchers identify insomnia patients with potentially rhythm-related maintaining factors and to provide structured hypotheses for phenotype-stratified randomized trials. Phenotype assignment should rely on multi-indicator convergence rather than a single marker, and no single diary, actigraphic, questionnaire-based, or wearable-derived variable should be considered sufficient for phenotype classification. A minimum clinical assessment set could include 1–2 weeks of sleep diary, workday-free day sleep midpoint differences, chronotype assessment, light exposure interview, evening screen use, daytime naps, caffeine and alcohol intake, medication use, psychiatric symptom screening, mood-state assessment, obstructive sleep apnea risk screening, and screening for other sleep disorders. Research-grade assessment can include actigraphy with light sensing, DLMO, core or skin temperature rhythms, PSG or ambulatory EEG, HPA-axis markers, daytime functioning, mood measures, and sleep regularity metrics such as the Sleep Regularity Index, interdaily stability, intradaily variability, and social jetlag (33, 34). This tiered structure separates feasible clinical assessment from mechanistic validation.

### 5.1 Provisional operational definitions for future research

To prevent candidate phenotypes from remaining purely conceptual, future studies should prespecify multi-indicator operational rules that combine symptom profiles, behavioral timing, objective rhythm markers, light exposure, psychiatric assessment, and screening for other sleep disorders. These provisional rules should not be treated as clinical diagnostic thresholds, but as research definitions to be tested for reliability, stability, predictive validity, and treatment-moderating value. A delayed-phase phenotype could require sleep-onset complaints with late habitual sleep timing, workday sleep restriction or morning sleepiness, delayed free-day sleep midpoint, increased workday-free day sleep midpoint discrepancy, and objective evidence of delayed DLMO or a shortened DLMO-to-target-bedtime phase angle. An advanced-phase phenotype could require early morning awakening accompanied by early evening sleepiness, an advanced habitual sleep window, early sleep midpoint, and support from advanced DLMO or core body temperature nadir, while carefully distinguishing circadian phase advance from depression-related early awakening, age-related sleep changes, medication effects, and COMISA-related sleep fragmentation. An unstable-rhythm phenotype could be defined by elevated day-to-day variability in sleep midpoint, increased workday-free day discrepancy, frequent catch-up sleep, elevated social jetlag, reduced Sleep Regularity Index, reduced interdaily stability, or increased intradaily variability over 1–2 weeks. A reduced-amplitude phenotype could combine low daytime activity, relatively high nocturnal activity, insufficient daytime light exposure, excessive nocturnal or sleep-period light exposure, reduced actigraphic relative amplitude, and, where available, attenuated melatonin, temperature, or cortisol rhythmicity. A hyperarousal-dominant phenotype should be assigned only when clear phase misalignment is absent and when sleep-related rumination, conditioned arousal, impaired sleep perception, pre-sleep cognitive-emotional activation, diary-actigraphy or diary-PSG discrepancy, psychiatric symptom assessment, medication review, and exclusion or stratification of other sleep disorders support this pathway. These definitions require validation for reliability, stability, and predictive validity across age groups, occupational schedules, cultures, and light environments.

Objective parameters that may support future phenotype classification include DLMO timing, DLMO-to-target-bedtime phase angle, core body temperature nadir, sleep midpoint and its day-to-day standard deviation, workday-free day sleep midpoint difference, social jetlag, Sleep Regularity Index, interdaily stability, intradaily variability, actigraphic relative amplitude, daytime activity level, nocturnal activity counts, daytime light exposure, sleep-period light exposure, and validated symptom measures such as insomnia severity, sleep-related rumination, depressive symptoms, anxiety symptoms, and bipolar-spectrum screening. These parameters should be interpreted jointly rather than hierarchically, because behavioral sleep timing, rest-activity rhythm, endogenous circadian phase, psychiatric status, and comorbid sleep disorders may diverge within the same patient. Even with such multi-indicator definitions, these phenotypes should be considered provisional research constructs until their inter-rater reliability, test-retest stability, longitudinal consistency, and treatment-moderating value have been examined prospectively.

For mixed presentations, sequencing of modules should follow three principles after screening for COMISA, active affective episodes, medication effects, substance use, and other sleep disorders. First, prioritize safe and feasible behavioral time cues such as fixed wake time, limiting catch-up sleep, regular daytime activity, and increasing morning natural light. Second, add phase-shifting interventions such as light or melatonin only when phase misalignment is supported by convergent evidence. Third, when hyperarousal, worry, rumination, or conditioned insomnia is prominent, CBT-I should remain central and circadian modules should be treated as adjunctive hypotheses.

## 6 Evidence strength and key limitations

Stronger evidence supports several foundational statements: sleep is jointly regulated by homeostatic and circadian processes; CRSWD can present with insomnia-like sleep-onset difficulty, early awakening, and daytime impairment; light and melatonin can shift circadian phase when timed appropriately; and CBT-I is first-line treatment for chronic insomnia disorder (8, 11, 38–44).

Moderate evidence concerns rhythm-related patterns within insomnia populations. Some patients with insomnia show delayed sleep timing, reduced rest-activity amplitude, increased sleep-timing variability, or abnormal light exposure; these characteristics have been associated with insomnia severity, daytime impairment, fatigue, mood symptoms, PSG variables, and psychiatric comorbidity (9, 10, 12–14, 28–33). However, current evidence does not establish that these abnormalities are causal in all patients or sufficient to guide treatment selection alone.

Hypothesis-stage evidence concerns whether reduced amplitude directly promotes hyperarousal, whether circadian misalignment contributes to cognitive or emotional impairment through REM instability, slow-wave sleep reduction, or impaired synaptic homeostasis, whether wearables can replace DLMO or core temperature rhythms for individualized treatment decisions, and whether closed-loop light, thermal, neurostimulation, or other digital interventions improve long-term insomnia outcomes (36, 37, 48–50).

The central limitation is the inferential distance between the proposed framework and the current direct evidence base in insomnia disorder. Although the framework is grounded in circadian biology and sleep research, several phenotype-intervention hypotheses are still derived from CRSWD research, healthy-adult circadian experiments, actigraphy-based associations, psychiatric-comorbidity samples, and indirect clinical evidence rather than from prospective phenotype-stratified trials in rigorously defined insomnia disorder cohorts. Consequently, the proposed phenotypes should not be assumed to be stable biological subtypes, and their reproducibility, temporal stability, incremental predictive value, and treatment-guiding utility remain unknown. First, this is a narrative review and did not involve independent dual screening, formal risk-of-bias assessment, or quantitative synthesis, so selective citation remains possible. Second, a large proportion of evidence comes from CRSWD, healthy circadian experiments, psychiatric comorbidity studies, or general circadian biology rather than from insomnia disorder samples. Third, candidate phenotypes lack standardized operational thresholds; their stability, reproducibility, predictive validity, and treatment-guiding value remain unknown.

Fourth, candidate phenotype classification may be confounded by COMISA, psychiatric comorbidities, medication effects, and substance use. These factors may also limit the generalizability of findings from mixed or psychiatric-comorbidity samples to uncomplicated insomnia disorder. Sleep fragmentation caused by respiratory events, mood-related early awakening, bipolar-spectrum sleep reduction, antidepressant- or sedative-related changes in sleep architecture, and anxiety-related pre-sleep arousal can mimic circadian phase delay, phase advance, rhythm instability, or reduced amplitude. Future studies should therefore prespecify whether such cases are excluded, stratified, or adjusted for in sensitivity analyses.

Fifth, circadian phenotyping is vulnerable to measurement error. DLMO requires controlled dim-light conditions, appropriate sampling time, posture control, and attention to diet and medications. Actigraphy captures behavioral rest-activity patterns and cannot separate endogenous phase delay from voluntary late sleep. In insomnia disorder, this limitation is particularly relevant because most actigraphic algorithms infer sleep from reduced movement. Quiet wakefulness, prolonged time in bed, and motionless rumination may therefore be scored as sleep, leading to overestimation of total sleep time and sleep efficiency and underestimation of wake after sleep onset. This may bias phenotype assignment when sleep timing, nocturnal activity, or rest-activity amplitude is derived primarily from actigraphy. Core body temperature and skin temperature are influenced by activity, food intake, ambient temperature, and measurement site. Light sensors are affected by device placement, viewing angle, occlusion, and adherence. Wearable devices remain limited in sleep staging, brief-awakening detection, endogenous phase estimation, algorithm transparency, and cross-device comparability. Long-term wearable monitoring also introduces adherence-related error, including non-wear time, device removal during sleep, battery charging, inconsistent placement, sensor occlusion, software or algorithm updates, and declining engagement over repeated weeks. Although wrist-worn actigraphy combined with mathematical models can predict DLMO or circadian misalignment in selected populations, errors may be clinically meaningful and generalizability across regular schedules, shift work, and rhythm-disordered populations requires further validation (49, 50). These errors can produce phenotype misclassification and attenuate detectable phenotype-response relationships. Future studies should combine actigraphy with sleep diaries, prespecified wear-time criteria, missing-data reporting, and, where feasible, PSG, DLMO, core temperature, or validated temperature-based markers to reduce misclassification.

Sixth, trials of circadian interventions have often been small, short, and insufficiently phenotype-stratified, and safety, adherence, burden, and cost-effectiveness remain under-studied.

## 7 Future directions

Future research should move from describing circadian abnormalities in insomnia-like presentations to testing whether candidate phenotypes are reproducible, stable over time, predictive of treatment response, and mechanistically linked to clinical improvement. A useful design is baseline circadian phenotyping, phenotype-stratified randomization, mechanistic mediation measurement, and long-term follow-up.

At baseline, studies should collect sleep diary data, actigraphy, light exposure, daytime activity, chronotype, and, when feasible, DLMO or core temperature rhythms, together with OSA risk assessment, psychiatric symptom measures, mood-state evaluation, medication review, and substance-use information. Patients can then be stratified by multi-indicator rules into delayed-phase, advanced-phase, reduced-amplitude, unstable-rhythm, hyperarousal-dominant, or mixed phenotypes. Within each stratum, standard CBT-I can be compared with CBT-I plus a phenotype-matched circadian module. Primary outcomes may include the Insomnia Severity Index and diary-derived sleep latency, wake after sleep onset, and sleep efficiency. Secondary outcomes should include DLMO phase, sleep midpoint, sleep regularity, daytime fatigue, mood symptoms, quality of life, treatment burden, adverse events, adherence, recurrence, and cost-effectiveness. Mediation analyses should determine whether phase advance, amplitude enhancement, or improved sleep regularity explains clinical benefit.

More feasible clinical rhythm assessment tools are also needed. DLMO and core temperature rhythms have high biological validity but can be costly and logistically difficult. Combined models using sleep diary, actigraphy, light exposure, skin temperature, heart-rate variability, and sleep regularity may help screen for likely circadian abnormalities, but such models must be validated against standard phase markers and must specify acceptable error margins.

Psychiatric comorbidity is a priority area because insomnia symptoms, circadian disruption, and affective symptoms may overlap phenomenologically and mechanistically. Depression may contribute to early morning awakening, reduced activity, fatigue, and apparent rhythm-amplitude reduction, whereas bipolar-spectrum disorders may involve reduced sleep need, delayed or irregular sleep timing, and vulnerability to mood destabilization during light exposure, melatonin use, or schedule shifts. Anxiety disorders, psychotic disorders, medication effects, and substance use may further modify sleep continuity, circadian timing, rest-activity rhythms, and treatment response. Future trials should therefore test whether circadian modules improve mood, cognition, and functioning beyond sleep symptoms while prespecifying psychiatric stratification, monitoring mood destabilization, and distinguishing circadian treatment response from improvement in psychiatric state. Implementation science and cost-effectiveness research are also essential: if circadian phenotyping is expensive, complex, burdensome, or difficult for clinicians to deliver, even theoretically attractive strategies may fail in routine care.

## 8 Conclusion

Insomnia disorder is mechanistically heterogeneous, and circadian regulation may contribute to symptom expression, maintenance, and treatment response in selected patients. The candidate phenotypes proposed in this review are intended to complement, rather than replace, established insomnia models centered on hyperarousal, sleep homeostasis, and cognitive-behavioral perpetuation.

At present, circadian phenotype matching remains a research direction within precision sleep medicine rather than a validated clinical pathway. Future studies should determine whether multi-indicator phenotype definitions are reliable, stable over time, predictive of treatment response, and associated with incremental benefit when circadian modules are added to CBT-I. Clinical implementation should await evidence for measurement validity, safety, feasibility, cost-effectiveness, and long-term outcomes.

## Data Availability

The original contributions presented in the study are included in the article/supplementary material. Further inquiries can be directed to the corresponding author.

## References

[1] Morin CM Benca R . Chronic insomnia. Lancet. (2012) 379:1129–41. doi: 10.1016/s0140-6736(11)60750-2 22265700

[2] Sateia MJ . International classification of sleep disorders. Chest. (2014) 146:1387–94. doi: 10.1016/b978-0-323-24288-2.00061-1 25367475

[3] Riemann D Espie CA Altena E Arnardottir ES Baglioni C Bassetti CLA . The European Insomnia Guideline: An update on the diagnosis and treatment of insomnia 2023. J Sleep Res. (2023) 32:e14035. doi: 10.1111/jsr.14035 38016484

[4] Ohayon MM . Epidemiology of insomnia: what we know and what we still need to learn. Sleep Med Rev. (2002) 6:97–111. doi: 10.1053/smrv.2002.0186 12531146

[5] Baglioni C Battagliese G Feige B Spiegelhalder K Nissen C Voderholzer U . Insomnia as a predictor of depression: a meta-analytic evaluation of longitudinal epidemiological studies. J Affect Disord. (2011) 135:10–9. doi: 10.1016/j.jad.2011.01.011 21300408

[6] Hertenstein E Feige B Gmeiner T Kienzler C Spiegelhalder K Johann A . Insomnia as a predictor of mental disorders: a systematic review and meta-analysis. Sleep Med Rev. (2019) 43:96–105. doi: 10.1016/j.smrv.2018.10.006 30537570

[7] Riemann D Spiegelhalder K Feige B Voderholzer U Berger M Perlis M . The hyperarousal model of insomnia: a review of the concept and its evidence. Sleep Med Rev. (2010) 14:19–31. doi: 10.1016/j.smrv.2009.04.002 19481481

[8] Borbély AA Daan S Wirz-Justice A Deboer T . The two-process model of sleep regulation: a reappraisal. J Sleep Res. (2016) 25:131–43. doi: 10.1111/jsr.12371 26762182

[9] Flynn-Evans EE Shekleton JA Miller B Epstein LJ Kirsch D Brogna LA . Circadian phase and phase angle disorders in primary insomnia. Sleep. (2017) 40. doi: 10.1093/sleep/zsx163 29029340

[10] Scott H Lovato N Comas M Bartlett D Grunstein RR Lack L . Circadian rhythm timing and associations with sleep symptoms in people with insomnia. J Pineal Res. (2025) 77:e70069. doi: 10.1111/jpi.70069 40761139 PMC12322714

[11] Auger RR Burgess HJ Emens JS Deriy LV Thomas SM Sharkey KM . Clinical practice guideline for the treatment of intrinsic circadian rhythm sleep-wake disorders: Advanced sleep-wake phase disorder (ASWPD), delayed sleep-wake phase disorder (DSWPD), non-24-hour sleep-wake rhythm disorder (N24SWD), and irregular sleep-wake rhythm disorder (ISWRD). An update for 2015: An American Academy of Sleep Medicine clinical practice guideline. J Clin Sleep Med. (2015) 11:1199–236. doi: 10.1007/978-3-030-43803-6_6 26414986 PMC4582061

[12] Ancoli-Israel S Cole R Alessi C Chambers M Moorcroft W Pollak CP . The role of actigraphy in the study of sleep and circadian rhythms. Sleep. (2003) 26:342–92. doi: 10.1093/sleep/26.3.342 12749557

[13] Smith MT Mccrae CS Cheung J Martin JL Harrod CG Heald JL . Use of actigraphy for the evaluation of sleep disorders and circadian rhythm sleep-wake disorders: An American Academy of Sleep Medicine clinical practice guideline. J Clin Sleep Med. (2018) 14:1231–7. doi: 10.5664/jcsm.7230 29991437 PMC6040807

[14] Meyer N Lok R Schmidt C Kyle SD Mcclung CA Cajochen C . The sleep-circadian interface: a window into mental disorders. Proc Natl Acad Sci USA. (2024) 121:e2214756121. doi: 10.1073/pnas.2214756121 38394243 PMC10907245

[15] Khalsa SB Jewett ME Cajochen C Czeisler CA . A phase response curve to single bright light pulses in human subjects. J Physiol. (2003) 549:945–52. doi: 10.1113/jphysiol.2003.040477 12717008 PMC2342968

[16] Cajochen C Münch M Kobialka S Kräuchi K Steiner R Oelhafen P . High sensitivity of human melatonin, alertness, thermoregulation, and heart rate to short wavelength light. J Clin Endocrinol Metab. (2005) 90:1311–6. doi: 10.1210/jc.2004-0957 15585546

[17] Duffy JF Wright KP Jr. Entrainment of the human circadian system by light. J Biol Rhythms. (2005) 20:326–38. doi: 10.1177/0748730405277983 16077152

[18] St Hilaire MA Gooley JJ Khalsa SB Kronauer RE Czeisler CA Lockley SW . Human phase response curve to a 1 h pulse of bright white light. J Physiol. (2012) 590:3035–45. doi: 10.1113/jphysiol.2012.227892 22547633 PMC3406389

[19] Chang AM Aeschbach D Duffy JF Czeisler CA . Evening use of light-emitting eReaders negatively affects sleep, circadian timing, and next-morning alertness. Proc Natl Acad Sci USA. (2015) 112:1232–7. doi: 10.1073/pnas.1418490112 25535358 PMC4313820

[20] Phillips AJK Vidafar P Burns AC Mcglashan EM Anderson C Rajaratnam SMW . High sensitivity and interindividual variability in the response of the human circadian system to evening light. Proc Natl Acad Sci USA. (2019) 116:12019–24. doi: 10.1073/pnas.1901824116 31138694 PMC6575863

[21] Chellappa SL . Individual differences in light sensitivity affect sleep and circadian rhythms. Sleep. (2021) 44. doi: 10.1093/sleep/zsaa214 33049062 PMC7879412

[22] Lok R Ancoli-Israel S Ensrud KE Redline S Stone KL Zeitzer JM . Timing of outdoor light exposure is associated with sleep-wake consolidation in community-dwelling older men. Front Sleep. (2023) 2:1268379. doi: 10.3389/frsle.2023.1268379 41426466 PMC12713814

[23] Mead MP Reid KJ Knutson KL . Night-to-night associations between light exposure and sleep health. J Sleep Res. (2023) 32:e13620. doi: 10.31219/osf.io/erjws 35599235 PMC9679040

[24] Littner M Kushida CA Anderson WM Bailey D Berry RB Davila DG . Practice parameters for the role of actigraphy in the study of sleep and circadian rhythms: an update for 2002. Sleep. (2003) 26:337–41. doi: 10.1093/sleep/26.3.337 12749556

[25] Benloucif S Burgess HJ Klerman EB Lewy AJ Middleton B Murphy PJ . Measuring melatonin in humans. J Clin Sleep Med. (2008) 4:66–9. doi: 10.5664/jcsm.27083 PMC2276833 18350967

[26] Carney CE Buysse DJ Ancoli-Israel S Edinger JD Krystal AD Lichstein KL . The consensus sleep diary: standardizing prospective sleep self-monitoring. Sleep. (2012) 35:287–302. doi: 10.5665/sleep.1642 22294820 PMC3250369

[27] Popovici C Steiropoulos P Mihaicuta S Dumitru S Dragila S Bikov A . The hidden burden of COMISA: Clinical implications and treatment challenges. Pulm Ther. (2026) 12:25–38. doi: 10.1007/s41030-025-00331-0 41219669 PMC12992872

[28] Roh HW Choi SJ Jo H Kim D Choi JG Son SJ . Associations of actigraphy derived rest activity patterns and circadian phase with clinical symptoms and polysomnographic parameters in chronic insomnia disorders. Sci Rep. (2022) 12:4895. doi: 10.1038/s41598-022-08899-2 35318367 PMC8941088

[29] Wittmann M Dinich J Merrow M Roenneberg T . Social jetlag: misalignment of biological and social time. Chronobiol Int. (2006) 23:497–509. doi: 10.1080/07420520500545979 16687322

[30] Roenneberg T Allebrandt KV Merrow M Vetter C . Social jetlag and obesity. Curr Biol. (2012) 22:939–43. doi: 10.1016/j.cub.2013.04.011 22578422

[31] Phillips AJK Clerx WM O'brien CS Sano A Barger LK Picard RW . Irregular sleep/wake patterns are associated with poorer academic performance and delayed circadian and sleep/wake timing. Sci Rep. (2017) 7:3216. doi: 10.1038/s41598-017-03171-4 28607474 PMC5468315

[32] Lunsford-Avery JR Engelhard MM Navar AM Kollins SH . Validation of the Sleep Regularity Index in older adults and associations with cardiometabolic risk. Sci Rep. (2018) 8:14158. doi: 10.1038/s41598-018-32402-5 30242174 PMC6154967

[33] Fischer D Klerman EB Phillips AJK . Measuring sleep regularity: theoretical properties and practical usage of existing metrics. Sleep. (2021) 44. doi: 10.1093/sleep/zsab103 33864369 PMC8503839

[34] Spitschan M Stefani O Blattner P Gronfier C Lockley SW Lucas RJ . How to report light exposure in human chronobiology and sleep research experiments. Clocks Sleep. (2019) 1:280–9. doi: 10.3390/clockssleep1030024 31281903 PMC6609447

[35] Xu YX Zhang JH Tao FB Sun Y . Association between exposure to light at night (LAN) and sleep problems: a systematic review and meta-analysis of observational studies. Sci Total Environ. (2023) 857:159303. doi: 10.1016/j.scitotenv.2022.159303 36228789

[36] Vgontzas AN Bixler EO Lin HM Prolo P Mastorakos G Vela-Bueno A . Chronic insomnia is associated with nyctohemeral activation of the hypothalamic-pituitary-adrenal axis: clinical implications. J Clin Endocrinol Metab. (2001) 86:3787–94. doi: 10.1210/jcem.86.8.7778 11502812

[37] Tononi G Cirelli C . Sleep and the price of plasticity: from synaptic and cellular homeostasis to memory consolidation and integration. Neuron. (2014) 81:12–34. doi: 10.1016/j.neuron.2013.12.025 24411729 PMC3921176

[38] Qaseem A Kansagara D Forciea MA Cooke M Denberg TD . Management of chronic insomnia disorder in adults: a clinical practice guideline from the American College of Physicians. Ann Intern Med. (2016) 165:125–33. doi: 10.7326/m15-2175 27136449

[39] Edinger JD Arnedt JT Bertisch SM Carney CE Harrington JJ Lichstein KL . Behavioral and psychological treatments for chronic insomnia disorder in adults: an American Academy of Sleep Medicine clinical practice guideline. J Clin Sleep Med. (2021) 17:255–62. doi: 10.5664/jcsm.8986 33164742 PMC7853203

[40] Trauer JM Qian MY Doyle JS Rajaratnam SM Cunnington D . Cognitive behavioral therapy for chronic insomnia: a systematic review and meta-analysis. Ann Intern Med. (2015) 163:191–204. doi: 10.7326/M14-2841 26054060

[41] Lewy AJ Bauer VK Ahmed S Thomas KH Cutler NL Singer CM . The human phase response curve (PRC) to melatonin is about 12 hours out of phase with the PRC to light. Chronobiol Int. (1998) 15:71–83. doi: 10.3109/07420529808998671 9493716

[42] Burgess HJ Revell VL Eastman CI . A three pulse phase response curve to three milligrams of melatonin in humans. J Physiol. (2008) 586:639–47. doi: 10.1113/jphysiol.2007.143180 18006583 PMC2375577

[43] Sateia MJ Buysse DJ Krystal AD Neubauer DN Heald JL . Clinical practice guideline for the pharmacologic treatment of chronic insomnia in adults: An American Academy of Sleep Medicine clinical practice guideline. J Clin Sleep Med. (2017) 13:307–49. doi: 10.1093/sleepj/zsx050.394 27998379 PMC5263087

[44] Van Geijlswijk IM Korzilius HP Smits MG . The use of exogenous melatonin in delayed sleep phase disorder: a meta-analysis. Sleep. (2010) 33:1605–14. doi: 10.1093/sleep/33.12.1605 21120122 PMC2982730

[45] Buysse DJ . Sleep health: can we define it? Does it matter? Sleep. (2014) 37:9–17. doi: 10.5665/sleep.3298 24470692 PMC3902880

[46] Harvey AG Dong L Hein K Yu SH Martinez AJ Gumport NB . A randomized controlled trial of the Transdiagnostic Intervention for Sleep and Circadian Dysfunction (TranS-C) to improve serious mental illness outcomes in a community setting. J Consult Clin Psychol. (2021) 89:537–50. doi: 10.1037/ccp0000650 34264701 PMC9377521

[47] Sarfan LD Hilmoe HE Gumport NB Harvey AG . The Transdiagnostic Intervention for Sleep and Circadian Dysfunction (TranS-C) in community mental health: Comorbidity and use of modules under the microscope. Cognit Behav Pract. (2023) 30:692–706. doi: 10.1016/j.cbpra.2022.03.007 39429752 PMC11488694

[48] De Zambotti M Cellini N Goldstone A Colrain IM Baker FC . Wearable sleep technology in clinical and research settings. Med Sci Sports Exerc. (2019) 51:1538–57. doi: 10.1249/mss.0000000000001947 30789439 PMC6579636

[49] Huang Y Mayer C Cheng P Siddula A Burgess HJ Drake C . Predicting circadian phase across populations: a comparison of mathematical models and wearable devices. Sleep. (2021) 44. doi: 10.1093/sleep/zsab126 34013347 PMC8503830

[50] Cheng P Walch O Huang Y Mayer C Sagong C Cuamatzi Castelan A . Predicting circadian misalignment with wearable technology: validation of wrist-worn actigraphy and photometry in night shift workers. Sleep. (2021) 44. doi: 10.1093/sleep/zsaa180 32918087 PMC8240654

